# Focused Electric Field Technology: A Novel Myoelectrical Stimulation Technology for Noninvasive Aging Muscle Rejuvenation

**DOI:** 10.1111/jocd.16749

**Published:** 2024-12-24

**Authors:** Boyang Jiang, Bingbing Chen, Jia Xu, Jieshu Luo, Min Yao

**Affiliations:** ^1^ FLOSSOM Shenzhen Research Institute Shenzhen China; ^2^ Shenzhen Rawskin Dermatology Shenzhen China; ^3^ Department of Plastic and Reconstructive Surgery Shanghai Ninth People's Hospital, Shanghai Jiao Tong University School of Medicine China Shanghai China

**Keywords:** clinical evaluation, muscle aging, muscle satellite cells, myoelectrical stimulation, myogenic regulatory family, wearable electronic devices

## Abstract

**Background:**

Myoelectrical stimulation improves muscle function and reduces muscle atrophy and aging. However, research on the mechanism underlying its cosmetic effect remains limited.

**Aims:**

The aim of this study was to evaluate the cosmetic effects of the myoelectrical stimulation provided by the wearable intelligent flexible beauty device and its focused electric field technology (FEFT) on facial skin and muscle rejuvenation.

**Patients/Methods:**

We conducted a single‐blind, randomized, self‐controlled clinical efficacy experiment on 31 female volunteers using the device. Using an FEFT‐based platform, mice with d‐galactose‐induced skeletal muscle aging were subjected to surface myoelectrical stimulation of the gastrocnemius. Immunohistochemical analysis of skeletal muscles and protein immunoblotting were used to analyze the effects of FEFT.

**Results:**

After 14 days of use, facial skin elasticity significantly increased, wrinkle firmness significantly decreased, and the lift height of the upper eyelid and eye corner angle significantly increased in the volunteers. Clinical evaluation showed improvements in the drooping of the upper eyelid and eye bags. Self‐evaluation questionnaires indicated alleviation of facial wrinkles. These improvements were more pronounced after 28 days. In mice, FEFT alleviated aging‐induced muscle fiber atrophy, muscle fiber cross‐sectional area reduction, and muscle satellite cell loss. FEFT also increased the expression of myogenic factors, including myogenic differentiation 1 (MYOD1).

**Conclusions:**

FEFT exerted a skin‐tightening effect by initiating myogenic processes and increasing the transformation of muscle satellite cells. Our research promotes the development of FEFT‐based medical rehabilitation or cosmetic anti‐aging products and provides a foundation for further application and comprehensive efficacy evaluation in human clinical settings.

## 1 Introduction

The physical manifestations of facial aging are now understood to include not only dermal aging, such as skin sagging and skin photoaging, but also the loss of deeper anatomy, such as fat volume, and the decline of muscle mass and function [1]. Although aging is a normal physiological process, skeletal muscle fibers have a certain degree of plasticity and muscle atrophy and loss can be reversed through muscle training [2]. Although resistance training (RT) is an effective means of combating neuromuscular decline, performing RT on specific delicate muscles, such as facial muscles, is difficult.

Recently, myoelectrical stimulation has been widely used in rehabilitation medicine [3]. The effects of facial muscle exercises on combating facial aging have been reported in previous studies [4]. A study demonstrated that continuous neuromuscular electrical stimulation applied to facial skin for 20 min/day over 12 weeks improved muscle stiffness, tension, and lift [5]. However, the mechanism underlying the cosmetic effect of myoelectrical stimulation requires further investigation.

Aging facial muscles are closely related to those affected by sarcopenia, a musculoskeletal disease that manifests as a loss of muscle mass and volume [1, 6]. During aging, the muscle fiber cross‐sectional area (CSA), which is highly correlated with muscle contraction force [7], decreases due to impaired muscle protein balance, denervation of muscle fibers, and a decrease in the number of muscle fibers. Facial aging is the result of the interaction of facial skeletal muscles, ligaments, adipose tissue, and skin changes, where skeletal muscle remodeling plays an important role [8]. Satellite cells are responsible for skeletal muscle remodelling [9]. The precursors of these myocytes are usually stationary and do not express myogenic regulatory factors, but when muscle tissues are damaged, certain satellite cells are activated and re‐enter the cell cycle, where they proliferate and produce new myoblasts.

An appropriate animal model for aging research with a focus on myoelectric stimulation is needed in our animal study. While natural aging models closely mimic human aging, they have the disadvantages of requiring extensive time and having low animal survival rates. On the other hand, induced aging models offer a shorter development period, easier application, and higher survival rates. For this experiment, we have chosen the d‐galactose‐induced skeletal muscle aging model to effectively control the degree of aging. Among the various induced aging models, d‐galactose is frequently utilized in aging studies due to its simplicity and relatively brief experimental duration. D‐galactose is a reducing sugar that exists naturally in the body and in a variety of foods. At high concentrations, its reduction product cannot be further metabolized by cells and accumulates in cells, resulting in cell dysfunction, metabolic disorders, and free radical accumulation. At the same time, the generated hydrogen peroxide can damage the lipid of cell membrane, increase the lipid peroxide and lipofuscin, etc., and the increased reactive oxygen species (ROS) generating in this process may subsequently lead to oxidative stress, inflammation, mitochondrial dysfunction and apoptosis, and then cause aging [10]. Studies conducted by Liao have found that d‐galactose intervention has significantly increased the markers of oxidative stress in the gastritis muscle of rats and mice, as well as the deficiency of mitochondrial complex I and the decline of muscle function [11].

The myogenic regulatory family (MRF) plays an important role in skeletal muscle remodeling and muscle generation. MRFs are a family of four basic helix–loop–helix transcription factors: myogenic factor 5 (*Myf5*), myogenic differentiation (*MyoD*), myogenin, and myogenic regulatory factor 4 (MRF4). *Myf5* is the first MRF to be embryonically expressed [12]. *MyoD* is expressed after *Myf5*. *Myf5* and *MyoD* are critical factors for myogenic cell determination [9]. A hierarchical relationship exists between the MRFs, shaping the dynamics of muscle remodeling and regeneration. Understanding these mechanisms is crucial for elucidating the pathophysiology of facial aging and developing interventions.

In this study, based on the results of an initial human efficacy test, we systematically examined the effects of myoelectrical stimulation using FEFT on myogenesis in mice with induced aging and studied how some myogenesis regulatory factors were affected. Our findings support the development of FEFT‐based medical and cosmetic products, paving the way for broader clinical applications and efficacy evaluations in humans.

## 2 Materials and Methods

### 2.1 Human Efficacy Test

Human efficacy tests were performed at the institute of Guangzhou Landproof Testing Technology Co. Ltd. (Guangzhou, China), and this study is an IRB‐approved study conducted to China and the approved number is *GDIRB* (*2024*)*4*–*1*. The benefits and risks of participation in the study were explained to each subject prior to entering the study, and then, all subjects need to sign informed consent.

Based on instrumental measurements, clinical evaluations, and participant self‐questionnaires, we recruited healthy Chinese female participants, aged 18 to 60 years, to evaluate the cutaneous acceptability and efficacy of the test products through a 28‐day period of human usage. Thirty‐three participants were enrolled after screening under detailed criteria of inclusion and exclusion (Tables S1–S3); 31 participants completed the entire trial, and 2 participants withdrew from the trial for personal reasons. The details of these participants are presented in Table 1.

**TABLE 1:** Information details on participants.

Number (person)	Minimum age (years old)	Maximum age (years old)	Mean age (years old)	Standard deviation (years old)
31	42	60	51.55	4.70

**FIGURE 1:**
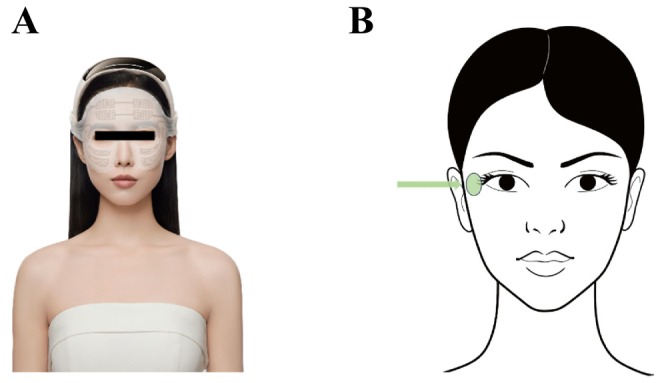
Application site of test product. Electrodes are mainly applied on periorbital area (A). Illustration of instrumental measurement analysis area (Green area with arrow) (B).

This study was designed as a single‐blind, randomized self‐comparison study, in which researchers and dermatologists are blinded from participants' information except their number with gender and age, while participants were recruited under criteria and numbered randomly, at the same time one side of each participant's face was chosen for testing randomly follow the random order table. Following the given method strictly, the test products were applied to participants (Table 2, Figure 1A). Self‐comparisons between the baseline value and measured value after using the product were conducted using a self‐questionnaire 14 and 28 days after consumer use of the FEFT device. The self‐questionnaire mainly evaluated feelings and satisfactions with the product's effect (Table 3). Skin elasticity (R2 and R5) and wrinkle firmness (F4) around the eyes were analyzed using a Cutometer MPA 580 (Courage+Khazaka electronic GmbH, Germany). The measurements were taken three times and averaged in the crow's feet area (Figure 1B). And upper eyelid lift height and eye‐corner angle were analyzed using a VISIA skin analysis imaging software (Canfield Scientific, USA). In addition, a clinical visual grade evaluation, including drooping of the upper outer eyelid and eye bag, was performed by dermatologists before and after 14‐ and 28‐day FEFT device usage. This clinical evaluation was based on the guidelines described in *SKIN AGING ATLAS VOLUME 2*: *ASIAN TYPE* [13].

**TABLE 2:** Details of product application steps.

Steps	Methods
1	After removing makeup and cleansing the face, we first place the sheet mask on the special eye mask tray and then pour the essence onto the sheet mask and use a small brush to evenly spread and saturate the entire eye mask
2	Take out the hair band controller, with the power button on the right side, wear the controller on the top of the head, both sides behind the ear
3	Long press the power button on the right side to turn on the device. It will enter standby mode with no current output
4	Then apply the electronic mask sheet onto the face, and gently press and smooth it with hands. We will pay attention to ensure that both sides of the cheeks are properly adhered to the skin without folding
5	Use the magnetic connectors on both sides of the electronic mask sheet to connect to the interfaces on the controller. When the connection is successful, the controller “beep” sounds and the green breathing light displays, indicating that the connection is successful
6	Press the power button briefly to start the current output at the Level 1. Continue pressing the power button to switch to the Level 2 or higher. At this time, two (Level 2) / three (Level 3) lights on the controller will illuminate, indicating the current output. Make sure that the indicator lights are on during each use
7	The treatment duration is 10 min. The controller will automatically stop when the time is up, indicating the end of the treatment, and daily skin care can be followed

**TABLE 3:** Analysis results of individual questionnaire.

No.	Question description	Time	Score						
			1	2	3	4	5	6	7
1	I feel that the product could reduce puffiness/swelling around the eyes after using the product	D14	0	0	0	0	0	19	12
2	I feel that the product could improve eye bags after using the product	D14	0	0	0	0	0	19	12
3	I feel that the product could diminish crow's feet after using the product	D14	0	0	0	0	1	18	12
4	I feel that the product could diminish fine lines under the eyes after using the product	D14	0	0	0	0	0	18	13
5	I feel that the product could diminish glabellar lines after using the product	D14	0	0	0	0	1	16	14
6	I feel that the product could improve lacrimal groove after using the product	D14	0	0	0	0	0	19	12
7	I feel that the product could improve skin firmness of the contours around the eyes after using the product	D14	0	0	0	0	0	17	14
8	I feel that the product could improve skin sagging around eyes after using the product	D14	0	0	0	0	0	18	13
9	I feel that the product could lift corners of eyes and improve drooping of upper eyelids after using the product	D14	0	0	0	0	0	17	14
10	I feel that the product could improve drooping eyebrows after using the product	D14	0	0	0	0	0	17	14
11	I feel that the product could lift the overall contour of the skin around the eyes after using the product	D14	0	0	0	0	0	18	13
12	I feel that the product could improve the fatigue around the eye after using the product	D14	0	0	0	0	0	16	15
13	I feel that the electronic mask enhances the absorption of the essence after using the product	D14	0	0	0	0	0	12	19
14	I did not feel any discomfort on my face, and the product is comfortable, mild and safe after using the product	D14	0	0	0	0	0	10	21
15	Overall, I am satisfied with the effectiveness of this product	D14	0	0	0	0	0	16	15
16	I feel that the product could reduce puffiness/swelling around the eyes after using the product	D28	0	0	0	0	0	6	25
17	I feel that the product could improve eye bags after using the product	D28	0	0	0	0	0	9	22
18	I feel that the product could diminish crow's feet after using the product	D28	0	0	0	0	0	8	23
19	I feel that the product could diminish fine lines under the eyes after using the product	D28	0	0	0	0	0	9	22
20	I feel that the product could diminish glabellar lines after using the product	D28	0	0	0	0	0	11	20
21	I feel that the product could improve lacrimal groove after using the product	D28	0	0	0	0	1	9	21
22	I feel that the product could improve skin firmness of the contours around the eyes after using the product	D28	0	0	0	0	0	5	26
23	I feel that the product could improve skin sagging around eyes after using the product	D28	0	0	0	0	0	7	24
24	I feel that the product could lift corners of eyes and improve drooping of upper eyelids after using the product	D28	0	0	0	0	0	6	25
25	I feel that the product could improve drooping eyebrows after using the product.	D28	0	0	0	0	0	8	23
26	I feel that the product could lift the overall contour of the skin around the eyes after using the product	D28	0	0	0	0	0	9	22
27	I feel that the product could improve the fatigue around the eye after using the product	D28	0	0	0	0	0	6	25
28	I feel that the electronic mask enhances the absorption of the essence after using the product	D28	0	0	0	0	0	5	26
29	I did not feel any discomfort on my face, and the product is comfortable, mild and safe after using the product	D28	0	0	0	0	0	2	29
30	Overall, I am satisfied with the effectiveness of this product	D28	0	0	0	0	0	4	27

*Note:* The subjects' feelings and effects of the FEFT products were rated on a scale of 1–7 (a score of 1 indicating strong disagreement or extreme dissatisfaction with the reported usage experience or test product's effectiveness, and a score of 7 indicating strong agreement or extreme satisfaction with the reported usage experience or test product's effectiveness, a score of 5 represents some level of agreement or satisfaction with the reported usage experience or test product's effectiveness. Higher scores indicate a greater level of affirmation or satisfaction with the reported usage experience or test product's effectiveness).

### 2.2 FEFT Device Details

The myoelectrical stimulation devices (FLOSSOM Intelligent wearable and flexible beauty devices, Lot: 2023080414004) were provided by FLOSSOM (GD) Beauty Technology Co. Ltd. The device has different version of sheet mask for different area on the face. Sheet mask version for periorbital usage was taken in this study (Figure 1A). The total size of the sheet is 4874 mm^2^, including 3290 mm^2^ electrode area. It can be stretched and adjusted moderately for a better fit in different participant.

**FIGURE 2:**
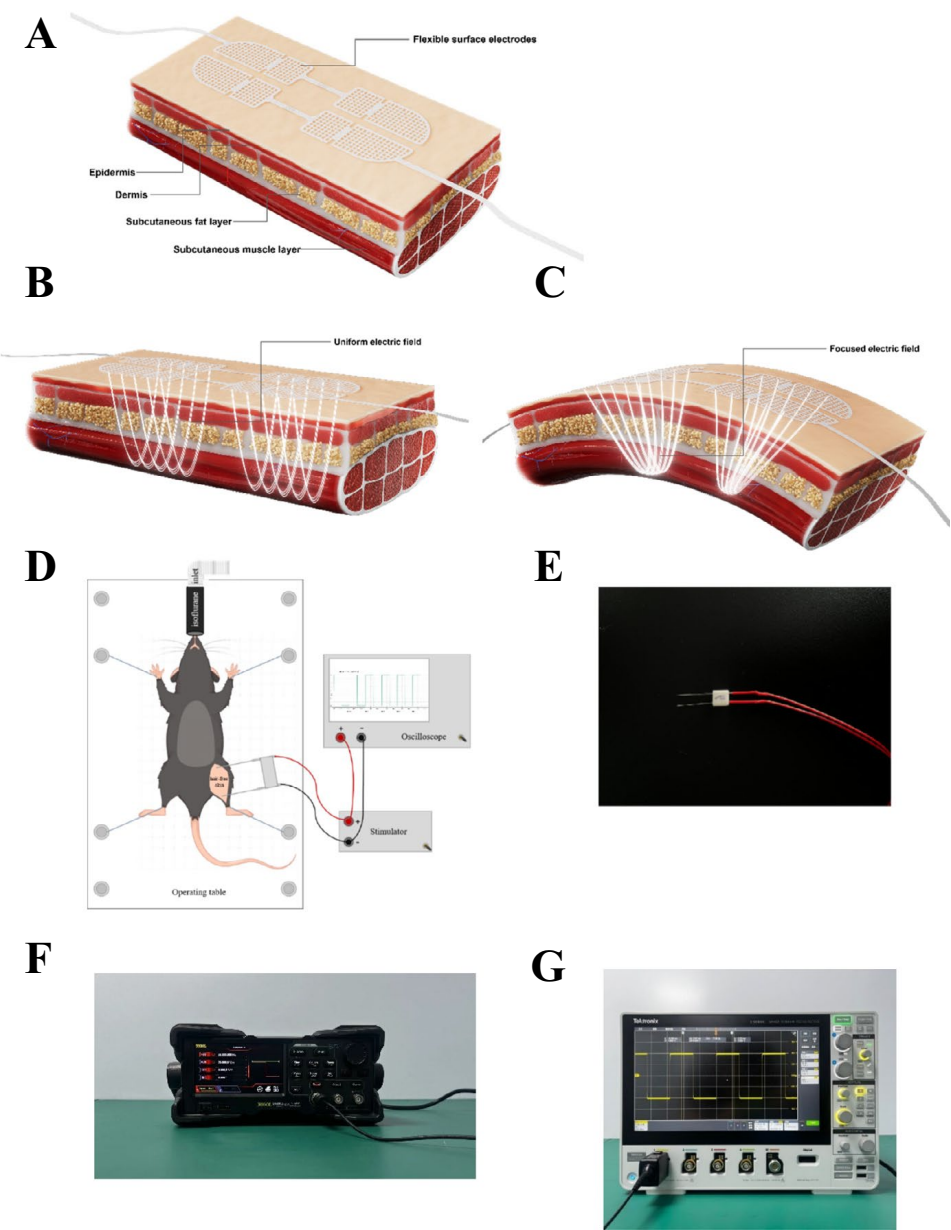
Myoelectrical stimulation system. In the human efficacy test, flexible surface electrodes were applied on subjects' face (A). When muscles are relatively flat and smooth, there are uniform electric fields (B). The flexible material texture helps the electrode slice completely fit the skin, making the stimulation of certain muscles more precise and stronger. They selectively induce contractions on a large scale of certain small delicate muscles in the face (C). Myoelectrical stimulation process of mouse experiment (D). Mice were fixed on the operating table with inhalation anesthesia, and their hairs on treated skin areas were removed with hair removal cream. Surface electrodes were from acupuncture needles with modifications (E). Stimulator (F). Oscilloscope (G).

FEFT is a novel myoelectrical stimulation technology for noninvasive aging muscle rejuvenation, specifically designed to selectively induce supramaximal contractions of small delicate muscles in the face. Compared with the large muscles of the limbs, the epidermis of facial skin naturally undulates because of the contours of facial bones and the presence of wrinkles. A previous study showed that most electrodes are flat and the electric field distribution is single, without focusing [14]. The flexible material texture of the FEFT causes the electrode slice to completely fit the skin, particularly facial muscles that naturally have a certain curvature, such as periocular muscles and the zygomaticus major, making the stimulation of certain muscles more precise and stronger (Figure 2A,B). They selectively induce large‐scale contractions in certain small, delicate muscles of the face (Figure 2C). The FEFT induces up to 88 energy impulses per second (88 Hz), which does not allow time for the facial muscles to relax between the individual signals. Appropriate selection of electric field strength and frequency results in maximal contraction.

### 2.3 Animal Model

Limited by facial biopsies from human tests, animal experiments can be used to further study the mechanism of myoelectrical stimulation in muscle rejuvenation. In this study, a d‐galactose‐induced skeletal muscle aging model was used to simulate aging in mice. The modeling dose and time of d‐galactose selected in this study were similar to those in the literature [10]. According to the methods of mouse and human age estimation reported in the literature [15], the degree of aging of the d‐galactose model in this experiment was equivalent to 40–50 years of human age, which was consistent with that of human efficacy test.

Eight‐week‐old male C57BL/6J mice were purchased from Guangdong Medical Laboratory Animal Center. All animals were maintained under 12‐h light and 12‐h dark cycles and were reared in incubators at 23°C with ad libitum feeding and drinking. After 1‐week acclimation, the mice were randomly divided into two groups: In one group, six mice were subcutaneously injected with phosphate‐buffered saline (PBS) as a normal control, and in the other group, 12 mice were injected with d‐galactose 600 mg/kg daily to serve as an aging animal model. After 6 weeks, the mice were randomly divided into two groups: aging control (OLD, *n* = 6) and electrical muscle stimulation for 2 weeks (OLD + FEFT, *n* = 6), with 5‐day treatment and 2‐day break in a week. PBS or d‐galactose treatment ended once FEFT treatment began. Surface electrodes were placed on the lower legs of the animals (Figure 2D). The mice in the aging group (sham control) were subjected to surface electrodes with the FEFT device turned off. The body weight gain was recorded weekly. After FEFT intervention, the mice were euthanized, and their gastrocnemius muscles were collected for further study. This study was an IRB‐approved study conducted in Guangdong Province of China and the approval number is *SYXK* (*YUE*) *2020–0230*.

### 2.4 Myoelectrical Stimulation System

**FIGURE 3:**
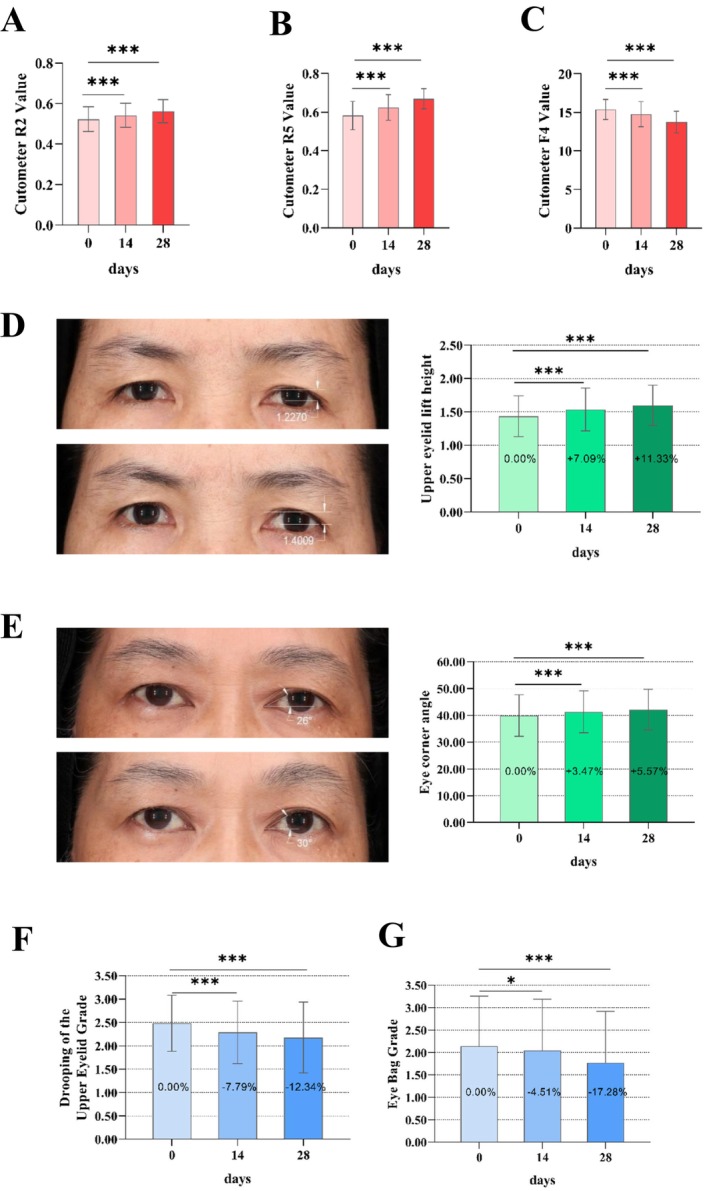
FEFT improves facial skin elasticity, firmness, and periocular muscle toning. Skin elasticity and firmness were measured by Cutometer, PA580. The parameters R2 (A) and R5 (B) were used as the basis for judging skin elasticity, and F4 (C) was used to evaluate skin firmness. The improvement of periocular muscle toning reflected in lift height of upper eyelid, eye corner angle, grading of drooping of the upper eyelid, and grading of eye bag. Lift height of upper eyelid was measured by capturing facial images using VISlA and analyzing with image analysis software (D). Eye corner angle was obtained by capturing facial images using VISIA and then analyzing with image analysis software (E). Drooping of the upper outer eyelid grade and eye bag grade were obtained by dermatologists (F and G). Evaluation was carried out as per standard image of “Drooping of the upper outer eyelid” grade of 0–6 (Table 1) and eye bag grade of 0–6 (Table 2). The lower the grade were, the lighter the drooping of the upper outer eyelid and the lighter eye bag would. Besides, individual questionnaires were performed and the results were shown in Table 3 and information details of all volunteers were shown in Table 4. Differences analysis between the baseline value (Day 0) and the measured values (Day 14 and Day 28) was performed by repeated‐measured ANOVA test, the sample size was 31. They were marked by *p* < 0.05* *p* < 0.01** and *p* < 0.001***.

The stimulation parameters for the FEFT device, as presented in Table 4, are based on the FLOSSOM wearable intelligent flexible beauty device. Because the size of the flexible electrodes of the FLOSSOM beauty device was not suitable for mice in this study, we recreated surface electrodes that were adapted to the lower legs of mice. The surface electrodes were acupuncture needles with some modifications (Figure 2E). As the figure showed, two acupuncture needles were used as electrodes and fixed in a certain distance. Needle tips would be in contact with the skin. The distance between the two electrodes varied for each mouse to ensure maximum muscle contractions. Needle ends were connected to positive wires and negative wires. Hair was removed using hair removal cream (Veet, France) before conductive gel (FLOSSOM, China) was applied to the skin surface. FEFT Stimulators were purchased from RIGOL (DG2052), and oscilloscopes were purchased from Tektronix (3 Series MDO34) (Figure 3F,G).

**TABLE 4:** FEFT parameters set in animal experiment.

Treat time	Frequency	Waveform	Duty cycle	Peak voltage
10 min	88 Hz	Biphasic symmetric square wave	50%	10 V

### 2.5 Antibodies

Primary antibodies targeting MYOD1 (ab203383) (Abcam, UK). Primary antibodies targeting MYF5 (SAB4501943) (Sigma‐Aldrich, USA). Primary antibodies targeting GAPDH (60004‐1‐Ig) (Protein Tech, USA). Primary antibodies were diluted in primary antibody diluent (P0023A) (Beyotime, China). Anti‐mouse (Cat.No.A0216) and anti‐rabbit (Cat.No.A0208) IgG horseradish peroxidase‐conjugated antibodies (Beyotime, China). Anti‐sarcomeric alpha‐actinin rabbit pAb (GB11555‐100) (ServiceBio, China). Secondary antibodies were diluted in 5% bovine serum albumin.

### 2.6 Histopathological Analysis

Treated muscles were isolated from mice as described previously [16]. Prefixing in 4% paraformaldehyde buffer, and then fixing in 10% formalin for 24 h. Dehydrating with gradient alcohol. 75% alcohol for 4 h, 85% alcohol for 2 h, 90% alcohol for 2 h, 95% alcohol for 1 h, anhydrous ethanol I for 30 min, anhydrous ethanol II for 30 min, alcohol benzene for 5~10 min, xylene II for 5~10 min, 65°C melting paraffin I for 1 h, 65°C melting paraffin II for 1 h, 65°C melting paraffin III for 1 h. Then embedding it in paraffin. The paraffin‐embedded samples were sectioned at 4‐μm thickness and stained with hematoxylin and eosin (H&E). Images were captured using a Leica Aperio Versa 200 microscope (Leica, Germany). Optical fields at 200× magnification within the tissue regions were observed. Referring to the literature, the CAS of muscle fibers was measured using the ImageJ software as described [17].

### 2.7 Quantitative PCR

Total RNA was extracted from the tissues using RNAiso plus reagent (Takara, Japan). Quantitative PCR (qPCR) was performed according to the manufacturers' instructions, as detailed as follows. Briefly, total RNA was reverse transcribed into cDNA using ReverTra Ace PCR RT Master Mix (Toyobo, USA). Subsequently, qPCR was performed using SYBR Premix Ex Taq II (Takara, Japan) and a connect instrument (Bioer, China) with LineGene 9600 Plus software. The following primers were used for qPCR: Mouse *MyoD1* forward, 5′‐CGG GAC ATA GAC TTG ACA GGC‐3′, and reverse, 5′‐TCG AAA CAC GGG TCA TCA TAG A‐3′ (83 bp); mouse *Gapdh* forward, 5′‐AGG TCG GTG TGA ACG GAT TTG‐3′, and reverse, 5′‐GGG GTC GTT GAT GGC AAC A‐3′ (95 bp); and mouse *Myf5* forward, 5′‐TGC CAG TTC TCC CCT TCT GA‐3′, and reverse, 5′‐AGG CTG CTA CTC TTG GCT CA‐3′, (97 bp). The following thermocycling conditions were used for qPCR: 95°C for 30 s and 40 cycles of 5 s at 95°C and 30 s at 56°C. Melting curve analysis of mRNA expression levels was quantified using the 2^−ΔΔCt^ method and normalized to the internal reference gene *Gapdh* using LineGene 9600 Plus software (Bioer instrument). Quantitative PCR was performed in duplicate.

### 2.8 Western Blotting

In this study, to measure MYOD1 and MYF5 protein expression levels, mice were first euthanized to obtain skeletal muscle tissues after different treatments. Mouse muscle tissue was ground in liquid nitrogen. Total protein was extracted from tissues using radioimmunoprecipitation assay buffer and quantified using a BCA Protein Assay kit (Beyotime, China). Proteins from skeletal muscles were subjected to sodium dodecyl sulphate‐polyacrylamide gel electrophoresis. The proteins were transferred to a polyvinylidene fluoride membrane (Millipore, United States). Membranes were blocked with 5% bovine serum albumin at room temperature for 1 h and incubated with primary antibodies at 4°C overnight. The membranes were then washed for four times, and each time lasted 5 min, and incubated with secondary antibodies at 37°C for 1 h. After washing the membrane for four times and each time lasted 5 min, we visualized the protein bands using automatic chemiluminescence image analysis system (Tanon 4600 SF) (Tanon, China).

### 2.9 Statistical Analysis

Statistical analyses were performed using GraphPad Prism Version 8.3. All results are expressed as mean ± standard deviation (SD). Comparisons between groups in animal experiments were performed using two‐tailed Student's *t*‐tests. Comparisons between baseline values (Day 0) and measured values (Days 14 and 28) of each parameter were analyzed using repeated‐measures analysis of variance (ANOVA) or the Wilcoxon signed‐rank tests. Statistical significance was defined as *p < 0.05, **p < 0.01, and ***p < 0.001.

## 3 Results

### 3.1 FEFT Improves Facial Skin Elasticity, Firmness, and Periocular Muscle Toning

Skin elasticity and firmness were measured using a shear instrument (MPA 580). Parameters R2 and R5 were used as the basis for evaluating skin elasticity. R2 refers to the ratio of rebounding without pressure to the maximum stretching under pressure, and R5 refers to the ratio of the elastic part of the skin during the recovery process to the elastic part of the skin during the pressure process. Greater values indicate better skin elasticity. Among the 31 volunteers, after using the wearable intelligent flexible beauty device, significant differences in skin elasticity (R2 and R5) of crow's feet at Days 14 and 28 were observed compared with the baseline value at Day 0 (*p* < 0.001; Figure 3A,B). The skin firmness (F4) was also measured using a Cutometer MPA 580. F4 is the area parameter obtained after 10 repetitions in test mode l, representing the degree of skin adsorption and stretching. Lower values indicate a stronger ability of the skin to resist deformation, that is, improved skin tightness. After applying the test product, significant decrease in the skin firmness (F4) of the crow's feet at each follow‐up time point were observed compared with the baseline value on Day 0 (Figure 3C).

Simultaneously, the lift height of the upper eyelid and the eye corner angles of the participants were analyzed. These parameters were measured by capturing facial images using VISIA and analyzing them using the image analysis software. After applying the FEFT device, compared with the baseline value, the lift height of the upper eyelid increased by 2.23%, 7.09%, and 11.33% on Days 0, 14, and 28, respectively (Figure 3D). Significant differences in the lift height of the upper eyelid were observed at Days 14 and 28 compared with the baseline value at Day 0 by repeated‐measures ANOVA (*p* < 0.05). As for the eye corner angle, compared with the baseline value, the eye corner angle increased by 3.47% and 5.57% on Days 14 and 28, respectively (Figure 3E). Significant differences in the eye corner angle at each follow‐up time compared with the baseline values on Day 0 were observed (*p* < 0.05).

Clinical evaluations were also performed by dermatologists using self‐questionnaires. Based on the *SKIN AGING ATLAS VOLUME 2*: *ASIAN TYPE* [13], drooping of the upper outer eyelid and eye bag grading were evaluated and analyzed (Figure 3F,G, Tables 5, 6, 7). The lower the grade were, the lighter the drooping of the upper outer eyelid and the lighter eye bag would be. The lower the grade were, the less severe the dark circles. Compared with the baseline value (Day 0), the drooping of the upper outer eyelid grade decreased by 7.79% and 12.34% on Days 14 and 28, respectively (Figure 2F). A significant alleviation of drooping of the upper outer eyelid was observed at each follow‐up time compared with the baseline value using the Wilcoxon signed‐rank test (*p* < 0.001; Figure 3F, Tables 5 and 7). In addition, compared with the baseline value (Day 0), the eye bag grade decreased 4.51% and 17.29% on Days 14 and 28, respectively (Figure 3G). The eye bag grade of the participants was significantly improved on Days 14 and 28 compared with the baseline value using the Wilcoxon signed‐rank test (*p* < 0.05; Figure 3G, Tables 6 and 7).

**TABLE 5:** Drooping of the upper outer eyelid grade atlas reference.

Grade	Standard atlas	Grade	Standard atlas	Grade	Standard atlas
0	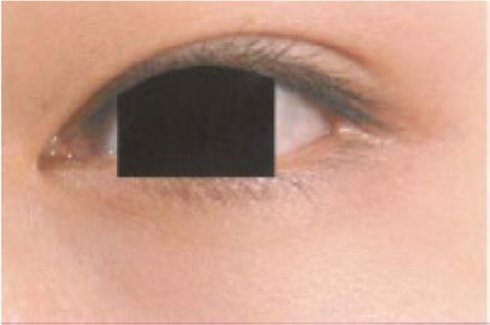	1	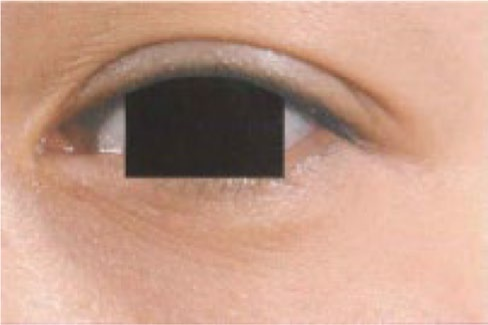	2	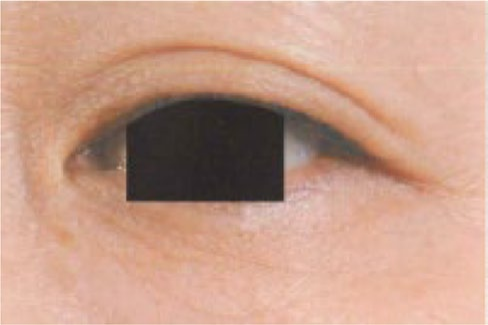
3	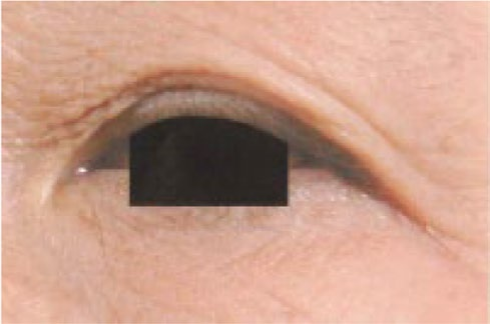	4	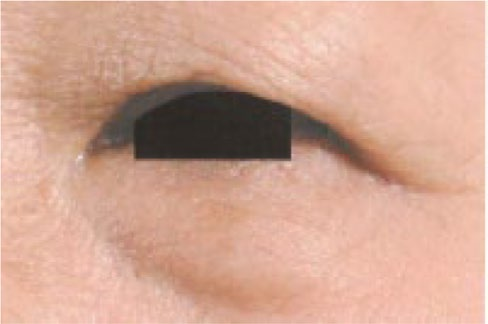	5	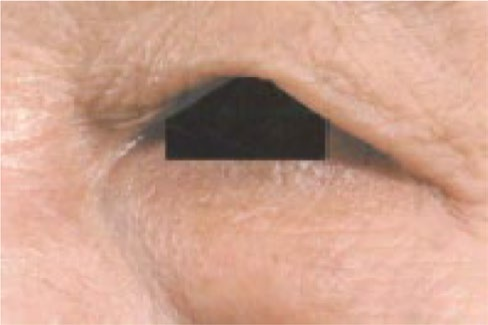
6	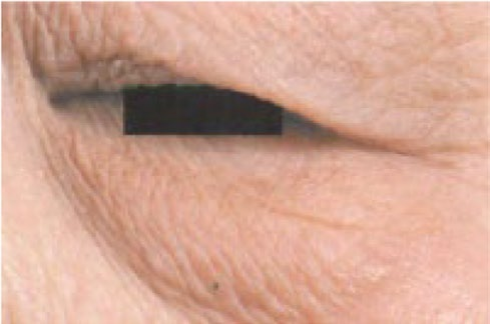				

**TABLE 6:** Eye bag grade atlas reference.

Grade	Standard atlas	Grade	Standard atlas	Grade	Standard atlas
0	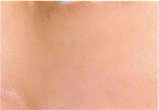	1	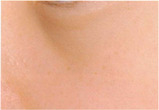	2	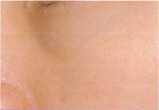
3	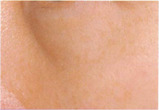	4	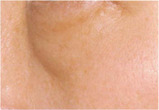	5	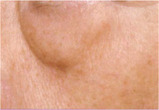
6	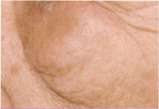				

**TABLE 7:** Analysis results of clinical evaluation.

Subject no.	Drooping of the upper outer eyelid grade evaluation			Eye bag grade evaluation		
	D0	D14	D28	D0	D14	D28
1	2.00	2.00	2.00	1.50	1.00	1.00
2	2.50	2.50	2.00	2.00	2.00	1.50
3	2.00	1.50	1.00	1.00	1.00	1.00
4	2.00	2.00	1.50	1.00	1.00	0.50
5	2.00	1.50	1.50	1.00	1.00	1.00
7	2.00	2.00	1.50	3.00	2.50	2.00
8	2.50	2.50	2.50	1.00	1.00	1.00
11	3.00	3.00	3.00	1.50	1.50	1.00
12	3.00	2.50	2.50	1.00	1.00	0.50
14	4.00	4.00	4.00	2.50	2.50	2.50
15	3.50	3.50	3.50	4.00	4.00	3.50
18	2.00	1.50	1.50	3.00	3.00	2.50
21	2.50	2.50	2.50	2.00	1.50	1.00
22	3.00	2.50	2.50	1.50	1.50	1.00
23	2.00	2.00	1.50	1.00	1.00	0.50
24	2.50	2.50	2.50	2.00	2.00	2.00
25	2.50	2.00	2.00	4.00	4.00	4.00
26	3.00	3.00	3.00	3.00	3.00	2.50
27	4.00	3.50	3.50	1.00	1.00	1.00
29	2.00	2.00	2.00	5.00	5.00	5.00
31	2.50	2.50	2.50	2.00	1.50	1.50
32	2.50	2.00	1.50	2.00	1.50	1.50
33	2.00	2.00	2.00	1.50	1.00	0.50
34	1.50	1.00	1.00	1.50	1.50	1.00
35	2.00	1.50	1.50	2.00	2.00	1.50
36	3.00	3.00	3.00	1.00	1.00	1.00
37	2.00	1.50	1.00	2.00	2.00	2.00
38	2.50	2.00	2.00	4.00	4.00	3.50
39	2.50	2.50	2.50	2.50	2.50	2.50
40	2.00	2.00	2.00	4.00	4.00	3.50
41	2.50	2.50	2.50	2.00	2.00	1.50
Mean	2.48	2.29	2.18	2.15	2.05	1.77
SD	0.60	0.67	0.76	1.11	1.14	1.15

A total of 31 participants completed a self‐questionnaire based on their own feelings and product effects on Days 14 and 28 using a 1–7 rating scale. A score of 1 indicates strong disagreement or extreme dissatisfaction with the reported usage experience or test product's effectiveness, and a score of 7 indicates strong agreement or extreme satisfaction with the reported usage experience or test product's effectiveness. A score of 5 represents some level of agreement or satisfaction with the reported usage experience or test product's effectiveness. Higher scores indicate a greater level of affirmation or satisfaction with the reported usage experience or the test product's effectiveness. Table 3 presents the questionnaire results obtained after using the test product. Almost all subjects gave a score greater than 5 for all questions after they used the device.

### 3.2 Modeling and Myoelectrical Stimulation Did Not Cause Significant Stress‐Related Weight Loss

**FIGURE 4:**
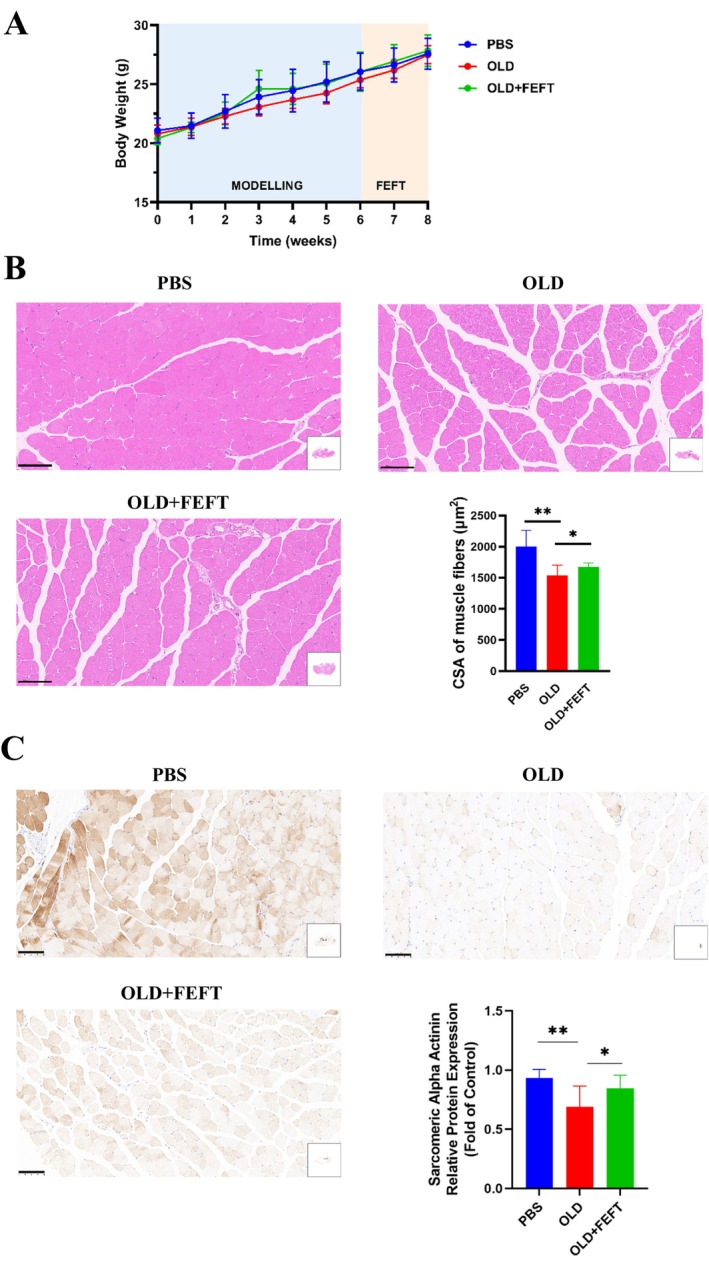
Modeling and myoelectrical stimulation did not cause significant stress weight loss (A), and FEFT alleviates D‐galactose‐induced muscle fiber atrophy and satellite cell loss. Histopathological sections of skeletal muscles (H&E), and statistical analysis of CSA of ten visual fields randomly picked from each group (B), scale bar: 100 μm. Localization and expression level of sarcomeric alpha actinin, and statistical analysis of protein expression in situ of ten visual fields randomly picked from each group (C), scale bar: 100 μm. Statistical analyses were performed using GraphPad Prism Version 8.3. All results are expressed as mean ± SD. Comparisons between groups in animal experiments were performed using Two‐tailed student *t*‐test. *p* < 0.05*and *p* < 0.01**.

During the animal experiment, a good mental state, smooth hair, free movement, uniform breathing, normal food intake, and normal fecal condition were observed in all groups (data not shown). No obvious skin damage was observed during the daily use of the apparatus. As shown in Figure 4A, the body weight of the mice in each group increased normally during each period of administration, and no significant difference in body weight compared with the model control group was observed (*p* > 0.05). Therefore, aging and electrical stimulation did not cause significant stress‐related weight loss in the tested animals.

### 3.3 FEFT Alleviates d‐Galactose‐Induced Muscle Fiber Atrophy and Satellite Cell Loss

Aging is accompanied by skeletal muscle fiber atrophy [18]. In this study, the successful induction of muscle fiber aging via subcutaneous administration of d‐galactose and whether myoelectrical stimulation exerted a beneficial effect on aging muscles were determined. After 2‐week myoelectrical stimulation intervention in an aging mouse model, the gastrocnemius and soleus muscles of mice from each group were immediately prepared for paraffin sectioning following euthanasia. As shown in Figure 4B, relative to the normal group, the muscle fiber structure in the aging group was loose and the muscle bundle size was different. In the myoelectrical stimulation group, relative to the model control group, the fibers were arranged more neatly, their structure was clearer, and the cytoplasmic staining of the muscle fibers was more uniform. As mentioned above, the CSA of skeletal muscles is highly correlated with muscle contraction force, which is closely related to the aging process [7]. Here, we analyzed the CSA of muscle fibers within these images in a semiquantitative manner using the ImageJ software [17]. The CSA in the aging group was significantly reduced compared to that in the normal group, and the CSA in the myoelectrical stimulation group was significantly increased compared to that in the aging group.

Studies showed that a decrease in the number of muscle satellite cells leads to muscle atrophy and loss [19] and that senescent muscular atrophy is related to a gradual decline in muscle activation, proliferation, and differentiation of myosatellite cells and their loss [9]. We therefore determined whether the proliferation and differentiation of muscle satellite cells in mice were consistent with our previous observations. Because sarcomeric alpha actinin is a myogenic marker expressed when myosatellite cells differentiate into myocytes [20], we used specific staining for sarcomeric alpha actinin to observe the proliferation and differentiation of satellite cells. The results showed that in the aging model group, the expression of sarcomeric alpha actinin decreased significantly compared to that in the normal control group (Figure 4C). After 2‐week myoelectrical stimulation, the expression of sarcomeric alpha actinin increased, which was in agreement with the histomorphology and changes in muscle structures observed in H&E‐stained sections.

### 3.4 FEFT Increases MyoD1 in Skeletal Muscles of Induced Aging Mice

**FIGURE 5:**
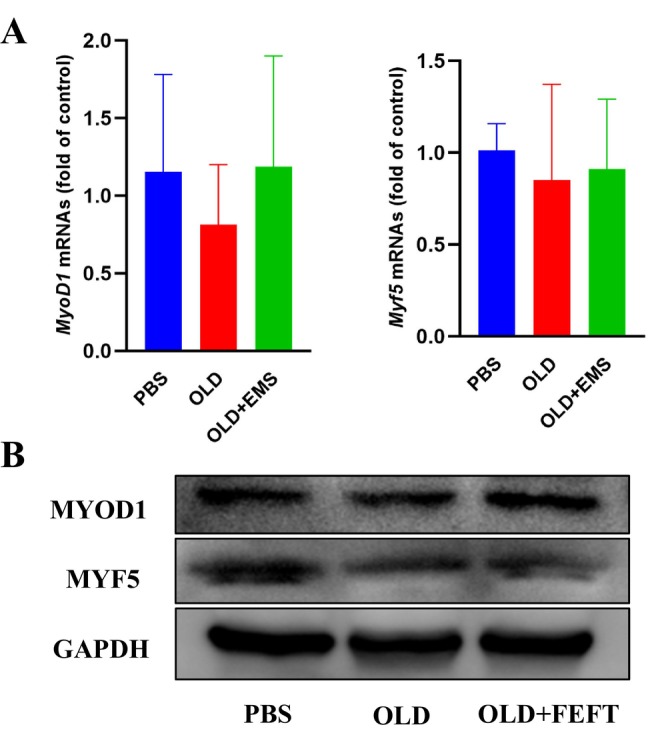
FEFT increases MYOD1 expression in skeletal muscles of induced aging mice. Changes of transcription level in *MyoD1* and *Myf5* (A), and expression levels of myogenesis‐related proteins MYOD1 and MYF5 (B).

Transcription levels of myogenesis markers in muscles were analyzed using qPCR. In the induced‐aging group, *MyoD1* and *Myf5* expression was significantly decreased (Figure 5A). After myoelectrical intervention, *MyoD1* and *Myf5* expression in the skeletal muscles showed an increasing trend but was not statistically significant.

The results of our histomorphological analysis suggest that FEFT effectively ameliorates skeletal muscle fiber atrophy during aging. Therefore, to further explore the possible mechanism by which FEFT affects skeletal muscle regeneration in mice, we extracted proteins from the gastrocnemius and soleus muscles of the mice in each group. The protein profiles of the muscles were analyzed by western blotting. In the induced‐aging group, MYOD1 expression decreased significantly (Figure 5B). After FEFT intervention, MYOD1 expression in skeletal muscles significantly increased, suggesting that the alleviation of muscle fiber atrophy in the electrical stimulation group may be related to an increase in MYOD1 expression. In addition, the myoelectrical stimulation group showed no significant increase in MYF5 expression in treated muscles.

## 4 Discussion

Myoelectrical stimulation has been found to provide health benefits in rehabilitation medicine and physical therapy, such as mitigating muscular degenerative changes and alleviating muscular pain. Myoelectrical stimulation has also been reported to assist in muscle strength and tone training [21]. In the present study, we systematically investigated the effects of FEFT devices on the facial skin of aging participants and skeletal muscle atrophy in mice with induced aging.

In the human efficacy test, our results showed that after using FEFT devices, skin elasticity and firmness were significantly improved. The lift height of the upper eyelid and eye corner angle showed an increasing trend on Days 14 and 28, which could be the result of increased resting facial muscle tone. Clinical evaluation by dermatologists and self‐questionnaires from volunteers showed the same improvement in the resting facial muscle tone and satisfaction with usage of the device. In the animal experiments, our results showed that when mice were administered d‐galactose for 4 weeks, FEFT intervention attenuated aging muscle atrophy, tightened the muscle fiber structure, and improved muscle satellite cell loss, similar to what was observed in another study [22] However, in certain cases, electrical stimulation causes mild skin redness at the site of the surface electrodes after treatment sessions, which is resolved within 10–20 min, consistent with what we observed in individuals during our experiment. This reaction to transcutaneous electrical stimulation is considered normal [23]. Nevertheless, unexpected adverse effects should still be considered when considering myoelectrical stimulation for muscle activation or rejuvenation.

Furthermore, the effect of FEFT on d‐galactose‐induced muscular aging was found not to be caused by changes in the stress conditions in mice. By restraining the apparatus assisted by isoflurane to perform respiratory anesthesia, the animal's state was steady with contraction of the treated muscles in the myoelectrical stimulation group during the entire intervention period. As we fixed the treated leg position of the mice during the intervention, every contraction could be considered muscle resistance training. The FEFT induces up to 88 energy impulses per second, which does not allow time for the facial muscles to relax between individual signals. Because the muscle is unable to relax, additional stimuli force the muscle to contract even further, which continuously builds up contraction power with every additional signal. The electrical field strength and frequency results in maximal contraction. However, little is known about the extent to which the facial muscles adapt to myoelectrical stimulation. Stimulation for 5 days with a 2‐day break may have offered the mice time to reduce muscle adaptation and muscle sensitivity to FEFT. As we described previously, CSA decreases during aging. Our histological results showed an increase in the CSA of muscle fibers in the stimulation group compared with the aging group, consistent with what is reported in the literature [24]. This showed an ability of muscular anti‐aging of FEET. In addition to CSA measurement for muscle aging evaluation in our study, some devices could have been used to determine resting muscle tone, for example, MyotonPRO. However, muscle strength training is time‐consuming. Thus, extending the time cycle of myoelectric stimulation to a certain load should be considered in future studies.

Because myoelectrical stimulation is essential for regulating myogenesis, we studied how FEFT modulates myogenesis in aging mice. Myogenic regulatory factors are positively associated with muscle development. Myoprogenitor cells follow a differentiation pathway regulated by myogenic regulatory factors [14]. Upregulation of the primary myogenic factor MYOD, a marker protein for the activation of muscle satellite cells, can promote skeletal muscle gene expression and initiate myogenic procedures to prevent muscular atrophy [25]. When mice were treated with d‐galactose for 4 weeks, MYOD1 expression significantly decreased. Conversely, FEFT intervention induced an increasing trend in MYOD1 expression, suggesting the activation of satellite cells that transformed myogenic progenitor cells into myoblasts and promoted the regeneration of skeletal muscle cells. MYF5 is thought to promote the expression of MYOD1 in skeletal muscles [12]. However, expression of *Myf5* showed no significant difference between the electrical intervention and sham control groups, though *Myf5* levels in the electrical intervention group showed an increasing trend compared to those in the aging modeling group. In this respect, some primary myogenic regulatory factors, such as MYF5, which respond to myoelectrical stimulation, may be in a quiescent state at the protein level. Certain studies have shown that during the progression of adult muscle satellite cells toward new muscle fiber formation, MYF5 is in a quiescent state, indicating that transcripts are present and have not yet been transcribed into proteins [12, 25]. Nevertheless, how exactly these alterations in electrical stimulation modulate MYOD1 and other factors involved in the later myogenic processes and whether secondary myogenic factors, such as myogenin, are involved in the proliferation and differentiation of satellite cells needs to be studied further.

In this study, myoelectrical stimulation altered the structure of skeletal muscles during muscle atrophy in aging mice, stimulating muscle reshaping and inducing a muscle hypertrophy reaction that reversed muscle atrophy. However, the characteristics of facial muscles of humans and mouse skeletal muscles may differ. Future studies are needed to determine the similarities between skeletal and facial muscles or to provide conclusive evidence that facial muscles respond similarly or differently to external stimuli. Besides, the current study focuses exclusively on Chinese females. Although the objective of our study was to examine muscle performance from three perspectives: molecular biology, animal models, and human subjects, further research involving a more diverse population should be considered for a comprehensive understanding.

In conclusion, based on a human efficacy test of FEFT devices, we monitored the effects of FEFT on facial skin and facial muscle toning and found that it significantly improved facial muscle toning. Animal experiments in aging mice were then conducted to investigate the underlying mechanisms of myoelectrical stimulation. Notably, FEFT alleviated d‐galactose‐induced aging in mice by increasing muscle activation and myogenesis via increased myogenic factor expression under treatment conditions. These results shed light on the mechanisms by which FEFT affects myogenic factor activation and myogenesis in humans. Our findings support the advancement of medical and cosmetic products utilizing FEFT, facilitating expanded clinical applications.

## Author Contributions

**Boyang Jiang:** methodology, investigation, validation, writing – original draft, writing – review and editing. **Bingbing Chen:** investigation, data curation, writing – original draft. **Jia Xu:** methodology, validation. **Jieshu Luo:** methodology, validation. **Min Yao:** conceptualization, writing – review and editing.

## Ethics Statement

All procedures were in accordance with the ethical standards of the responsible committee on human experimentation (institutional and national) and with the Helsinki Declaration of 1975, as revised in 2000. Informed consent was obtained from all patients included in the study. Animal experimental manipulations were undertaken in accordance with the Institutional Guidelines for the Care and Use of Laboratory Animals, Department of Science and Technology of Guangdong Province (Permit number SYXK (Yue) 2020–0230), and were performed in accordance with the NIH Guide for the Care and Use of Laboratory Animals.

## Conflicts of Interest

The authors declare no conflicts of interest.

## Supporting information


Table S1.
Table S2.
Table S3.

## Data Availability

The data that support the findings of this study are available on request from the corresponding author. The data are not publicly available due to privacy or ethical restrictions.
