# Supplementary material for: Focused Electric Field Technology: A Novel Myoelectrical Stimulation Technology for Noninvasive Aging Muscle Rejuvenation
Source: J Cosmet Dermatol. 2024 Dec 24;24(2):e16749. doi: 10.1111/jocd.16749 (PMC11845949; doi:10.1111/jocd.16749)
Supplement: Supplementary file 1 — Table S1. Table S2. Table S3. [file JOCD-24-e16749-s001.docx]

**S.Table 1.** Information details on participant selection criteria

| **NO.** | **Description** |
| --- | --- |
| 1 | Healthy Chinese female, aged from 18 to 60 years old. |
| 2 | The skin around the eyes lacks elasticity, the skin is sagging. |
| 3 | Visible crow's feet, underneath eye wrinkles and lacrimal groove wrinkles around the eyes along with drooping of the upper eyelids. |
| 4 | Localized darkening skin around the eyes is more obvious, with dark circles and eye bags, |
| 5 | Participants can well meet the program researcher's requirements and sign the Information Consent Forms. |
| 6 | Participants can well cooperate with the test project and reflect her own physical conditions, medication, and adverse reaction symptoms timely. |

**S.Table 2.** Information details on exclusion criteria

| **NO.** | **Description** |
| --- | --- |
| 1 | Those who are currently pregnant, breast feeding or planning pregnancy within 2 months. |
| 2 | Those with obvious sunburn, scar, hyperpigmentation and hairiness on test sites or other factors that may affect the test results. |
| 3 | Those with test sites which was infected with bacteria, viruses or fungi. |
| 4 | Those with any chronic dermatitis (e.g. skin tumors, acne rosacea, eczema, lupus erythematosus, seborrheic dermatitis, psoriasis and severe exfoliation, etc.). |
| 5 | Those with a history of immunosuppression/immune deficiency disorders (including HIV infection or AlDS) or currently using immunosuppressive medications and/or radiation. |
| 6 | Those with any uncontrolled disease such as asthma, epilepsy, diabetes, hypertension, hyperthyroidism or hypothyroidism or endocrine diseases; |
| 7 | Those who participated in other clinical trials within 3 months. |
| 8 | Current or recent (within previous 6 months) using any medically prescribed products that might affect skin condition or reactivity, such as anti-histamines, antibiotics, insulin, anti-inflammatory agents, Vitamin A, steroids, corticosteroids, aspirin, thyroid medication, etc.. |
| 9 | Those with medical cosmetology including skin cosmetic treatment such laser, chemical peeling and minimally invasive cosmetic treatments such as Botox on the test site within 6 months. |
| 10 | Those who have been diagnosed with allergies or suspected allergic reactions (systemic, inhaled or topical), or are allergic to ingredients of the formula. |
| 11 | There are other lesions or condition that reduce the possibility of enrollment or make enrollment complex, such as frequently changes in working environment and unstable living environment, which are easy to cause loss of follow up alcoholic or psychoactive substances, drugs abusers and dependents. |
| 12 | Those with mental illness or unable to take care of oneself. |

**S.Table 3.** Information details on exit criterion

| **NO.** | **Description** |
| --- | --- |
| 1 | If participants suffered from adverse events, missed follow-up without any reasons, contravened the test program (such as using other cosmetics or drugs that would affect test results) or other special reasons, after being confirmed not suitable to continue participating in this project by test organization, the subject would be required to withdraw. |
